# Efficacy and Safety of Electroacupuncture Through Nerve Stimulation in Patients With Anxiety Disorders: Protocol for a Randomized, Assessor-Blind, Three-Arm, Parallel-Group Clinical Trial

**DOI:** 10.2196/68166

**Published:** 2025-07-21

**Authors:** In Chul Jung, Dong-Hoon Kang, Sunyoung Choi, Yujin Choi, Ojin Kwon, Hye Jeong Kook, Daeun Lee, Yang-Chun Park, Jieun Kim

**Affiliations:** 1 Department of Oriental Neuropsychiatry College of Korean Medicine Daejeon University Daejeon Republic of Korea; 2 KM Science Research Division Korea Institute of Oriental Medicine Daejeon Republic of Korea; 3 Clinical Trial Center Daejeon Korean Medicine Hospital of Daejeon University Daejeon Republic of Korea; 4 Department of Internal Medicine College of Korean Medicine Daejeon University Daejeon Republic of Korea

**Keywords:** anxiety disorders, electroacupuncture, nerve stimulation, vagus nerve, median nerve

## Abstract

**Background:**

Anxiety disorders are among the most common mental health disorders, affecting a significant portion of the population. However, conventional treatments, such as pharmacotherapy and psychotherapy, often have limited effectiveness and may lead to undesirable side effects. Consequently, there is a growing demand for new alternative treatments for anxiety disorders. Recent studies suggest that electroacupuncture may demonstrate therapeutic effects in managing anxiety by mediating nerve stimulation.

**Objective:**

This study is designed to assess the efficacy and safety of electroacupuncture in treating anxiety disorders through nerve stimulation. Specifically, it will involve stimulating the median nerve at the PC6 acupoint (Neiguan) and the vagus nerve at the TF4 acupoint (Shenmen of ear acupuncture).

**Methods:**

This study is a randomized, assessor-blind, three-arm, parallel-group clinical trial comprising the PC6 electroacupuncture group, TF4 electroacupuncture group, and a control group. Participants will include patients diagnosed with social anxiety disorder, panic disorder, agoraphobia, and generalized anxiety disorder. Eligible participants will be randomly assigned to one of the 3 groups, with each group containing 20 individuals. The electroacupuncture groups will receive treatments at the designated acupoints twice weekly for 8 weeks, totaling 16 sessions. The control group will receive usual care without any treatment interventions through the end of the study period. The primary outcome is the comparison of Hamilton Anxiety Rating Scale scores between the treatment groups and the control group. Secondary outcomes include scores on the Hamilton Anxiety Rating Scale, Beck Anxiety Inventory, Beck Depression Inventory-II, Patient Health Questionnaire-15, World Health Organization Quality of Life Assessment Instrument abbreviated version, Penn State Worry Questionnaire, Panic Disorder Severity Scale, and Leibowitz Social Anxiety Scale. Safety evaluation variables include the frequency of adverse events, vital signs, and suicide risk assessment. Exploratory variables include performance on the Emotional Reactivity Test, empathy quotient, and heart rate variability.

**Results:**

The first participant was enrolled on December 15, 2022. As of October 2024, a total of 60 participants have been fully registered, and the intervention is currently in progress. We expect the completion of this trial to occur within the year 2025.

**Conclusions:**

In this study, we will evaluate the safety and efficacy of electroacupuncture for anxiety disorders. By elucidating the therapeutic mechanisms of electroacupuncture through nerve stimulation, this study will provide clinical evidence to support the development of potential interventions for patients with anxiety disorders.

**Trial Registration:**

Clinical Research Information Service of the Republic of Korea KCT0008378; https://cris.nih.go.kr/cris/search/detailSearch.do/24503

**International Registered Report Identifier (IRRID):**

DERR1-10.2196/68166

## Introduction

Anxiety disorders are defined as mental health conditions that are characterized by a set of symptoms, including excessive fear, anxiety, and related behavioral disturbances [1]. Anxiety disorders represent a significant proportion of mental health conditions, affecting an estimated 301 million people worldwide as of 2019 [2]. The prevalence of anxiety disorders is increasing due to factors such as an aging population, urbanization, and socioeconomic development [3]. These disorders not only result in substantial socioeconomic consequences but also pose a threat to the ongoing growth and productivity of societies [4]. Therefore, the treatment and management of anxiety disorders are becoming increasingly important.

Treatment for anxiety disorders has been found to be effective with pharmacotherapy such as antidepressants and benzodiazepines [5], and psychotherapies like cognitive behavioral therapy and eye movement desensitization and reprocessing are also used [6,7]. However, pharmacotherapy raises concern due to side effects and resistance [8,9], and is often met with negative perceptions by patients regarding psychiatric medications [10]. In addition, although psychotherapy is generally effective, it is neither always cost-effective nor consistently successful [11,12]. Thus, there is a need for treatments that are both cost-effective and associated with fewer adverse effects.

Recent studies suggest that acupuncture and electroacupuncture are effective treatments for anxiety disorders with fewer adverse effects compared to conventional pharmacotherapy [13,14]. Consequently, both acupuncture and electroacupuncture are being recommended as alternative methods for the chronic management and long-term treatment of anxiety disorders [15].

This study aims to assess the efficacy and safety of electroacupuncture treatment for anxiety disorders, with a particular focus on treatments at acupoints PC6 (Neiguan) and TF4 (Shenmen of ear acupuncture), compared with a control group.

## Methods

Study Design

**Figure 1:**
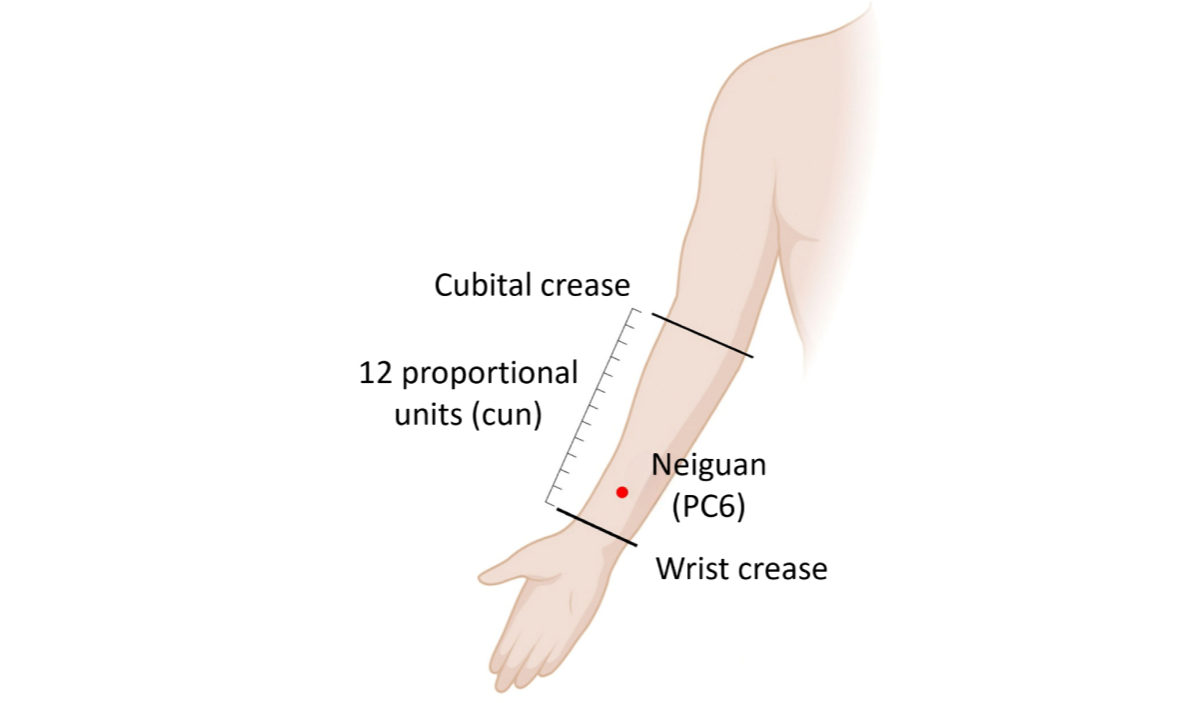
Acupoint PC6. Adapted from Li et al [16], under the Creative Commons Attribution 4.0 International License (CC BY 4.0).

**Figure 2:**
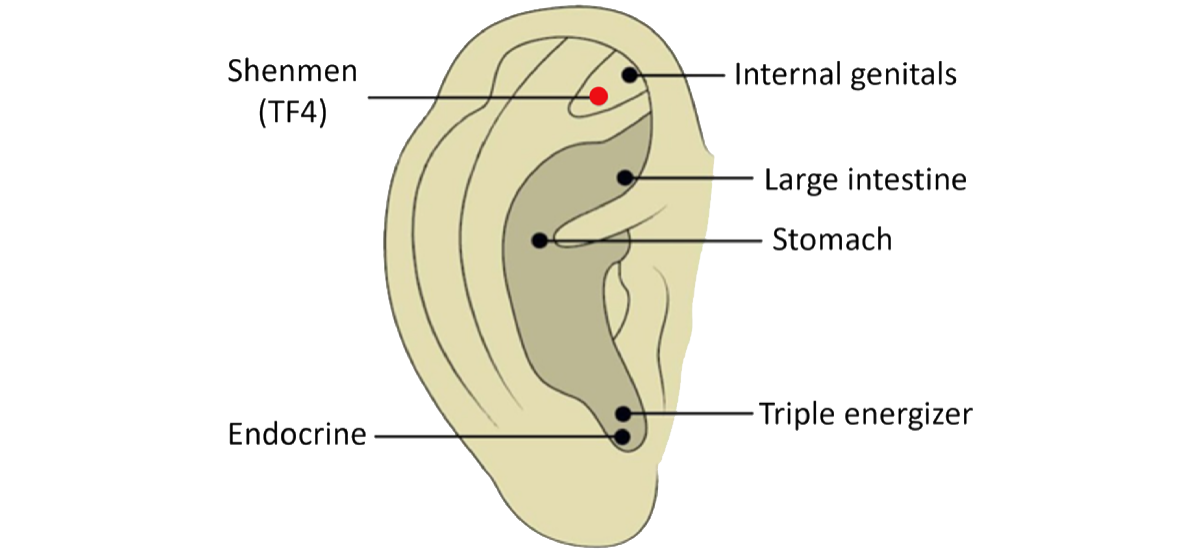
Acupoint TF4. Adapted from Qu et al [17], under the Creative Commons Attribution 4.0 International License (CC BY 4.0).

This study is designed as a randomized, 3-arm assessor-blinded, parallel-group clinical trial, which will be conducted at the Daejeon Korean Medicine Hospital of Daejeon University, Republic of Korea. It aims to evaluate the efficacy and safety of 2 distinct electroacupuncture interventions in the treatment of anxiety disorders, in comparison to usual care. The interventions specifically target to stimulate the median nerve via acupoint PC6 and the vagus nerve via acupoint TF4 (Figures 1 and 2) [16,17]. The control group, constituting usual care, will not receive any treatment.

To ensure the rigor and validity of our findings, this study protocol (version 1.4, June 2023) adheres to the SPIRIT (Standard Protocol Items: Recommendations for Interventional Trials) guidelines.

Study Procedure

During the initial screening visit, potential participants will receive a comprehensive explanation of the study’s purpose, procedures, risks, and benefits and will be asked to provide written consent. Those who voluntarily consent and meet the inclusion and exclusion criteria—as determined through demographic information surveys, medical and medication history reviews, vital signs measurements (blood pressure, pulse, and temperature), laboratory tests, pregnancy tests, the Structured Clinical Interview for *DSM-5* (*Diagnostic and Statistical Manual of Mental Disorders* [Fifth Edition]; SCID-5) [18], and the Columbia Suicide Severity Rating Scale (C-SSRS) [19]—may be enrolled in this study. The SCID-5 will be used to diagnose subtypes of anxiety disorders, including social anxiety disorder, panic disorder, agoraphobia, generalized anxiety disorder, and other specified anxiety disorders. Participants who do not meet the diagnostic criteria for an anxiety disorder as per the SCID-5 will be excluded from the study. The C-SSRS will be used to assess the risk of suicide; participants scoring 4 or above, indicating a high risk of suicide, will also be excluded from study selection.

Following registration, participants will be randomly assigned to one of the three groups: the electroacupuncture-vagus nerve stimulation (EA-VNS) group, the electroacupuncture-median nerve stimulation (EA-MNS) group, or the usual care group. Participants in the treatment groups will undergo electroacupuncture sessions twice weekly for a total of 16 sessions over 8 weeks. Conversely, the usual care group will not receive any interventions during this period.

**Figure 3:**
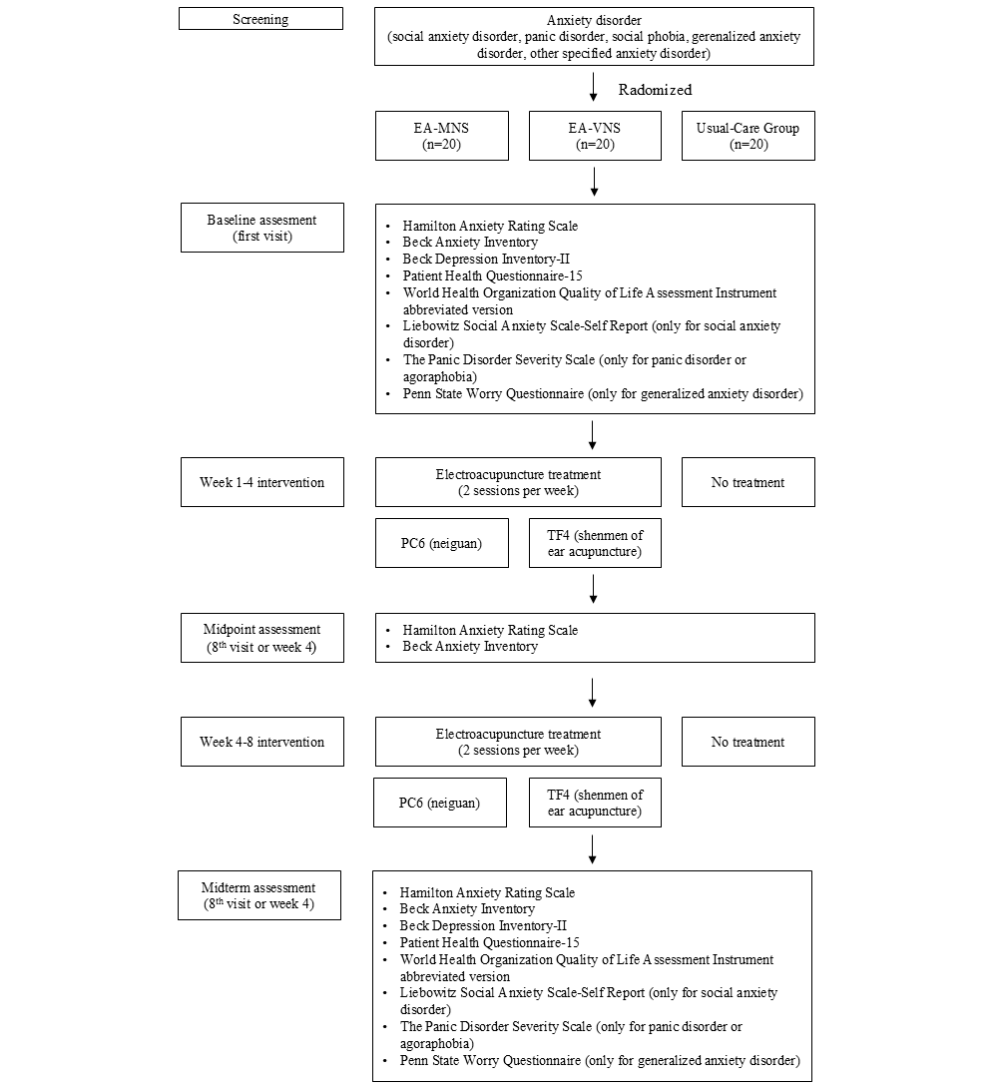
Flowchart of study procedure. EA-MNS: electroacupuncture-median nerve stimulation; EA-VNS: electroacupuncture-vagus nerve stimulation;.

The evaluation of the treatment group is scheduled to occur at the initial, eighth, and final visits. For the control group, assessments will be conducted in the first, fourth, and eighth weeks. During the initial and final assessments for the treatment group, and the first and eighth weeks for the control group, the following instruments will be administered: Hamilton Anxiety Rating Scale (HAM-A) [20], Beck Anxiety Inventory (BAI) [21], Beck Depression Inventory-II (BDI-II) [22], Patient Health Questionnaire-15 (PHQ-15) [23], World Health Organization Quality of Life Assessment Instrument abbreviated version (WHOQOL-BREF) [24], Leibowitz Social Anxiety Scale-Self Report (LSAS-SR) [25], The Panic Disorder Severity Scale (PDSS) [26], and Penn State Worry Questionnaire (PSWQ) [27]. LSAS-SR will be conducted only for patients with social anxiety disorder, PDSS only for those with panic disorder or agoraphobia, and PSWQ only for those with generalized anxiety disorder. A midpoint evaluation using the HAM-A and BAI will be conducted during the eighth visit for the treatment group or in the fourth week for the control group. The detailed schedules are described in Table 1 and the study flow is presented in Figure 3.

**Table 1:** Timeline of the trial.

Period				Screening (week –1)		Treatment									
						Baseline (week 0)	Midterm						Final (week 8)		
							Weeks 1-4		Week 4		Week 5-8				
Visit						Visit 1^a^	Visit 2-7^b,c^		Visit 8		Visit 9-15		Visit 16		
Consent				✓											
Demographic information				✓											
Medical history				✓		✓	✓		✓		✓		✓		
Physical examination				✓											
Vital signs				✓		✓	✓		✓		✓		✓		
Body weight and height				✓									✓		
Laboratory test^d^				✓											
Pregnancy test^e^				✓											
SCID-5^f^				✓											
C-SSRS^g^				✓									✓		
Inclusion and exclusion criteria				✓											
Randomization						✓									
	Item														
		HAM-A^h^, BAI^i^				✓			✓				✓		
		PHQ-15^j^, BDI-II^k^				✓									
		WHOQOL-BREF^l^				✓							✓		
		PSWQ^m^, PDSS^n^, LSAS-SR^o^				✓							✓		
		EQ^p^, PSS^q^				✓							✓		
Emotional reactivity test						✓							✓		
Check concomitant drugs						✓	✓		✓		✓		✓		
Check adverse effects						✓	✓		✓		✓		✓		
EA-MNS^r^, EA-VNS^s^						✓	✓		✓		✓		✓		
HRV^t^						✓			✓				✓		
Education of visit schedule				✓		✓	✓		✓		✓				

^a^Visit 1 is scheduled to occur within 10 days of screening.

^b^Visits 2-7 are implemented 4 times within 2 weeks + 2 days from visit 1. Similarly, visits 9- 15 are implemented 4 times within 2 weeks + 2 days after visit 8. Electroacupuncture treatments are typically scheduled twice a week, but once to three visits per week are permitted.

^c^Only the experimental group follows this schedule.

^d^Lab test items are as follows: complete blood cell count (red blood cell, white blood cell, hemoglobin, hematocrit, platelet, erythrocyte sedimentation rate, white blood cell differential count), blood chemistry test (aspartate aminotransferase, alanine aminotransferase, blood urea nitrogen, creatinine, and hemoglobin A_1c_), thyroid function test (triiodothyronine, free thyroxine, and thyroid stimulating hormone), pregnancy test (urine human chorionic gonadotropin, applicable only to fertile women).

^e^A pregnancy test is only performed for fertile women who have had a menstrual period within the last year.

^f^SCID-5: Structured Clinical Interview for DSM-5 (Diagnostic and Statistical Manual of Mental Disorders [Fifth Edition]).

^g^C-SSRS: Columbia Suicide Severity Rating Scale.

^h^HAM-A: Hamilton Anxiety Rating Scale.

^i^BAI: Beck Anxiety Inventory.

^j^PHQ-15: Patient Health Questionnaire-15.

^k^BDI-II: Beck Depression Inventory-II.

^l^WHOQOL-BREF: World Health Organization Quality of Life Assessment Instrument abbreviated version.

^m^PSWQ: Penn State Worry Questionnaire. This has been used for generalized anxiety disorder and other anxiety disorders.

^n^PDSS: Panic Disorder Severity Scale.

^o^LSAS-SR: Leibowitz Social Anxiety Scale-Self Report. This has been for social anxiety disorder.

^p^EQ: Empathy Quotient.

^q^PSS: Perceived Stress Scale.

^r^EA-MNS: electroacupuncture-median nerve stimulation. This will be conducted according to each group.

^s^EA-VNS: electroacupuncture-vagus nerve stimulation. This will be conducted according to each group.

^t^HRV: heart rate variability.

### Study Populations

To ensure the safety of participants and the integrity of study outcomes, specific inclusion and exclusion criteria were applied. These criteria were designed to select individuals with appropriate clinical characteristics for the study intervention while minimizing potential risks and confounding variables. Eligible participants were required to meet all inclusion criteria and none of the exclusion criteria listed in Textbox 1.

**Inclusion and exclusion criteria.**

Inclusion criteria:

(1) Aged 19-70 years, as individuals aged 19 years and older are legally permitted to provide autonomous consent to participate in clinical research in Korea.

(2) Diagnosed with an anxiety disorder including social anxiety disorder, panic disorder, agoraphobia, generalized anxiety disorder, other specified anxiety disorders, and unspecified anxiety disorder as assessed through the Structured Clinical Interview for *DSM-5* (*Diagnostic and Statistical Manual of Mental Disorders* [Fifth Edition]; SCID-5).

(3) Voluntarily decided to participate in this study and signed the written consent form.

(4) Able to complete electroacupuncture-neural stimulation treatments over 8 weeks.

Exclusion criteria:

(1) At high risk of suicide as assessed by the Columbia-Suicide Severity Rating Scale with a score of 4 or more.

(2) With a history of schizophrenia or bipolar disorder.

(3) Diagnosed with separation anxiety disorder, selective mutism, substance-induced anxiety disorder, anxiety disorder due to medication, or anxiety disorder due to other medical conditions.

(4) Received acupuncture treatment within the last 4 weeks.

(5) With a history of alcohol or other substance use disorders within the last 8 weeks.

(6) With a history of cerebrovascular disease, brain tumor, or traumatic brain injury.

(7) With a history of neurological or systemic disease that may affect the central nervous system.

(8) In medical conditions requiring inpatient treatment.

(9) Usage of a pacemaker.

(10) With contraindications to acupuncture treatment (eg, acupuncture-associated vasovagal response or tissue damage due to acupuncture).

(11) Taken medication or participated in another clinical trial within the last month.

(12) Pregnant or lactating women, or those not using medically acceptable methods of contraception during the study period.

(13) Deemed inappropriate for enrollment due to other reasons as determined by the investigators.

### Interventions

In this study, electroacupuncture treatments will be administered by qualified Korean medical doctors with at least 1 year of clinical experience. Participants assigned to the electroacupuncture treatment groups will receive treatments twice a week, with a total of 16 sessions over an 8-week period. Each session will last 20 minutes. For the EA-MNS group, the needle will be inserted at PC6, while for the EA-VNS group, the needle will be inserted at TF4. The selection of these intervention sites is based on previous studies showing that stimulation of the median nerve or PC6 acupoint and the vegus nerve or TF4 acupoint is effective for alleviating anxiety symptoms [15,28,29].

The needles will be stimulated using an electroacupuncture stimulator (model STN-110, Streatek Co). For the electroacupuncture-median nerve stimulating group, needles will be inserted at PC6 and at a nonacupoint approximately 0.5 cm proximal to PC6 on the right arm. Subsequently, the hook-shaped or magnetic output terminal of the electroacupuncture stimulator will be attached to administer electrical stimulation. The electrical stimulation for EA-MNS will be conducted at 12 Hz for 20 minutes. For the electroacupuncture-vagus nerve stimulating group, needles will be inserted at TF4 and at a nonacupoint surrounding 0.7 to 1.0 cm away from TF4 on the right ear. The method of attaching the electroacupuncture stimulator is the same as for the EA-MNS group. The electrical stimulation for EA-VNS will be conducted at 100 Hz for 20 minutes. In both groups, the intensity of the electrical stimulation (in mA) will be set to a level that the participant reports as a 4 to 5 on a scale from 0 to 10. The Standards for Reporting Interventions in Clinical Trials of Acupuncture (STRICTA) for acupuncture treatment is described in Multimedia Appendix 1.

### Criteria for Concomitant Drugs

#### Permissible Drugs

Drugs that have been taken consistently at a stable dose for at least 4 weeks before study participation and are not expected to interfere with the interpretation of the clinical trial results may be permitted at the discretion of the researcher. In addition, drugs intended for the transient treatment of other conditions can be coadministered after consultation with the researcher. Comprehensive details of all concomitant drugs—including the name of the drug, purpose of administration, dosage, and duration—will be documented in the case report form.

#### Prohibited Drugs

During the trial period, the administration of drugs that may influence anxiety symptoms is prohibited, including systemic steroids, antipsychotics, antidepressants, benzodiazepines, and sleeping pills. However, if antidepressants, benzodiazepines, and sleeping pills have been administered at a stable dose for at least 2 weeks before screening, their continuous use may be permitted.

### Efficacy Variables

The primary, secondary outcome measures, and research hypotheses are summarized in Textbox 2.

**Efficacy variables.**

**Primary Outcome**

The primary outcome will be assessed by analyzing changes in the Hamilton Anxiety Rating Scale (HAM-A) scores from baseline to week 8 in two separate comparisons:

- Between the electroacupuncture-median nerve stimulation (EA-MNS) group and the Usual Care group.
- Between the electroacupuncture-vagus nerve stimulation (EA-VNS) group and the Usual Care group.

Research Hypothesis

- Hypothesis 1 [H0 : μ1 = μ2, H1 : μ1 ≠ μ2]
  μ1: Changes in HAM-A score from baseline to week 8 in the EA-MNS group
  μ2: Changes in HAM-A score from baseline to week 8 in the Usual Care group
- Hypothesis 2 [H0 : μ3 = μ2, H1 : μ3 ≠ μ2]
  μ2: Changes in HAM-A score from baseline to week 8 in the Usual Care group
  μ3: Changes in HAM-A score from baseline to week 8 in the EA-VNS group

**Secondary Outcomes**

The secondary outcomes will be assessed as follows:

- Changes in HAM-A scores from baseline to week 8 between the EA-MNS group and the EA-VNS group.
- Evaluation of rates of loss of diagnosis and complete remission of anxiety disorder, as measured by HAM-A scores, from baseline to week 8 between the EA-MNS group and the Usual Care group.
- Evaluation of rates of loss of diagnosis and complete remission of anxiety disorder, as measured by HAM-A scores, from baseline to week 8 between the EA-VNS group and the Usual Care group.
- Evaluation of rates of loss of diagnosis and complete remission of anxiety disorder, as measured by HAM-A scores, from baseline to week 8 between the EA-MNS group and the EA-VNS group.
- Changes in Beck Anxiety Inventory (BAI), Beck Depression Inventory-II (BDI-II), Patient Health Questionnaire-15 (PHQ-15), World Health Organization Quality of Life Assessment Instrument abbreviated version (WHOQOL-BREF), Penn State Worry Questionnaire (PSWQ), Panic Disorder Severity Scale (PDSS), and Leibowitz Social Anxiety Scale-Self Report (LSAS-SR) scores from baseline to week 8 between the EA-MNS group and the Usual Care group.
- Changes in BAI, BDI-II, PHQ-15, WHOQOL-BREF, PSWQ, PDSS, and LSAS-SR scores from baseline to week 8 between the EA-VNS group and the Usual Care group.
- Changes in BAI, BDI-II, PHQ-15, WHOQOL-BREF, PSWQ, PDSS, and LSAS-SR scores from baseline to week 8 between the EA-MNS group and the EA-VNS group.

### Safety Variables

The safety evaluation variables in this study will include the frequency of adverse events, vital signs, and suicide risk assessment. All adverse events that occur during the clinical trial will be assessed for their severity and the causal relationship with the intervention, and their frequency will be used as an evaluation variable. If clinically significant adverse events are observed, changes in vital signs will be assessed compared to preintervention values. The C-SSRS will be used to evaluate the risk of suicide in participants, and a score of 4 or higher will be considered indicative of a suicide risk.

### Exploratory Variables

The exploratory variables in this study will include the Emotional Reactivity Test, Empathy Quotient (EQ), Perceived Stress Scale (PSS), and Heart Rate Variability (HRV). The Emotional Reactivity Test, EQ, and PSS will be assessed at baseline and at week 8, while HRV will be assessed at baseline, week 4, and week 8.

The Emotional Reactivity Test is a behavioral assessment that measures an individual’s responses to both positive and negative emotional stimuli. This test is designed to assess the phenomenon of emotional contagion by observing changes in facial expressions in response to emotional stimuli. It is based on experimental models from past research on emotion processing [30,31]. Participants are shown video clips that comprise 4 emotional contexts (joy, sadness, fear, anxiety, and neutral) and their facial expressions are recorded while they watch the emotional stimulus clips. Following the recording phase, participants will report the degree of their emotional state in response to the emotional stimuli on a scale of 0-9 using E-Prime software (Psychology Software Tools). Facial expressions will be analyzed using iMotion software (iMotions A/S).

The electrocardiography signal will be recorded using electrodes placed on the participant’s chest. Heart rate and R-R intervals will be extracted from the electrocardiography data using an in-house script developed in MATLAB (2016b, The MathWorks, Inc). The time-domain HRV metrics will include the mean R-R interval, the SD of R-R interval, and the root-mean-square of successive differences. The frequency-domain HRV metrics that will be calculated include the low-frequency power (0.04-0.15 Hz), high-frequency power (0.15-0.4 Hz), and the normalized low- and high-frequency power following standard recommendations [32].

### Sample Size

Considering the absence of prior clinical trials proving the therapeutic effect of electroacupuncture in anxiety disorders compared to a control group, this study references preliminary research recommending a minimum of 15 participants per group to ensure feasibility and facilitate exploratory analyses [33]. Therefore, to accommodate potential variances and ensure statistical robustness, we plan to enroll 15 participants per group, for a total of 45 participants. However, to enhance statistical rigor and account for potential attrition, the final sample size was adjusted to 20 participants per group, yielding a total of 60 participants.

### Randomization and Allocation

In this study, participants will be randomly assigned to one of the 3 groups: the EA-VNS group, the EA-MNS group, or the usual care group. Stratified block randomization will be used, using SAS software (version 9.4 or higher, SAS Institute), to ensure an unbiased and equal allocation (1:1:1 ratio) across the groups. The stratification criteria will include the specific type of anxiety disorder diagnosed in each participant, such as panic disorder, agoraphobia, generalized anxiety disorder, social anxiety disorder, and other specified anxiety disorders. This method ensures proportional representation of each anxiety disorder subtype within each study arm, thereby controlling for variability in treatment response based on the type of anxiety disorder. The randomization list in this study will be held by an independent statistician and will not be disclosed to ensure confidentiality. The randomized codes will be placed in opaque, sealed envelopes and stored in a locked cabinet. Randomization envelopes will be opened in the order of participant enrollment to assign each group. The opened envelopes will be stored separately, and the date, time of opening, and the opener’s signature will be recorded on the envelopes.

### Blinding

Given the nature of the interventions, it is not feasible to blind participants in this study. However, to preserve the integrity of the study outcomes, assessor blinding will be strictly maintained. Researchers responsible for evaluating the efficacy and safety outcomes will not participate in the administration of interventions or the randomization process.

### Data Management

All clinical trial-related information, including case report forms, consent forms, and supporting documentation, will be recorded, processed, and preserved to facilitate proper reporting, interpretation, and verification. Records containing participants' personal information will be strictly confidential. In all documents associated with the clinical trial, including case report forms, participants will be identified only by their identification codes and initials, not by their names.

### Monitoring

Monitoring will be conducted to ensure that the clinical trial is executed in accordance with the approved protocol and applicable regulations. This process includes verifying compliance with the protocol, ensuring accurate and appropriate data collection, reviewing consent and re-consent forms, and verifying the proper collection and reporting of adverse events.

### Statistical Methods: Definition of the Analysis Set

The analysis sets for evaluating data obtained in this clinical trial are defined as follows: the Full Analysis Set is in accordance with to the intent-to-treat principle, encompassing all participants who met the inclusion and exclusion criteria, were randomly assigned, and for whom baseline measurements and at least 1 postbaseline primary outcome was obtained. The Per Protocol Set (PPS) includes participants from the FAS who have adhered to at least 75% of the total intervention and do not meet any discontinuation or dropout criteria. The Safety Analysis Set (SAS) comprises all participants who have undergone at least one safety assessment following random assignment.

All data, including efficacy outcomes, will be analyzed using the FAS, with the PPS serving as a secondary analysis group for additional analyses. Safety variables will be analyzed using SAS. Continuous data will be presented as means and confidence intervals, while categorical data will be presented as frequencies and percentages (%). If necessary, initial characteristics of participants measured at screening or baseline may be categorized for subgroup analysis.

Unless specifically stated otherwise, all statistical analyses in this study's results will be conducted as 2-sided tests, with a significance level set at 5%. However, for the analysis of the primary outcome, the significance level will be set at 2.5%. Statistical analyses will be performed using SAS software (version 9.4 or higher, SAS Institute).

### Statistical Methods for Analyzing Efficacy Outcomes

#### Primary Outcomes

The primary outcome analysis will use a mixed-effect model repeated measure (MMRM) approach, including each treatment group and visit time point as fixed factors, with participants as random factors, and accounting for interactions between groups and visit time points. Variables that show statistically significant differences in demographic or sociological characteristics, or other factors potentially influencing anxiety disorders, will be incorporated as fixed factors. Results will be presented with means, 97.5% CIs, and *P* values. In addition, if significant baseline differences exist between groups, an Analysis of Covariance (ANCOVA), adjusted for baseline values as a covariate, will be conducted.

#### Secondary Outcomes

The analysis of secondary outcome variables will adhere to the same methodology used for the primary outcomes. Changes in continuous variables from baseline to week 8 in BDI-II, BAI, PHQ-15, WHOQOL-BREF, PSWQ, PDSS, and LSAS-SR will be assessed using the same analytical approach, with a significance level set at 5%. To evaluate the differences in measurements before and after the intervention within each group, the Student Paired *t* test or Wilcoxon signed rank test will be used for both primary and secondary outcomes. Additionally, to examine the trend changes over time between the EA-MNS and EA-VNS groups, Repeated Measures ANOVA will be conducted. This analysis will include testing the interaction between the groups and visits to ascertain how differences evolve over time. If the interaction is found to be significant, post hoc tests will be implemented to identify significant differences between the groups at specific time points based on baseline measurements. Furthermore, the binary variables such as loss of diagnosis and complete remission of anxiety disorder, as assessed by HAM-A, will be compared between groups using logistic regression, which will provide odds ratios and 95% CI.

### Statistical Methods for Safety Outcomes

The safety analysis will encompass the severity of adverse events, their causal relationship with the intervention, the incidence rate of adverse events by group, the rate of adverse events leading to dropout, and the incidence rate of serious adverse events. The proportion of participants who dropped out due to treatment failure or adverse events will be reported for each group, along with 95% CI. In addition, the chi-square test or Fisher exact test may be used to compare observed frequencies against expected frequencies. Also, the number of cases assessed as having a high risk of suicide during the study period will be presented by group, and statistical testing may be conducted if necessary.

### Statistical Methods to Handle Missing Data

When using the MMRM for variable analysis, no separate imputation for missing values will be conducted. Conversely, in cases where ANCOVA is used, missing values will be imputed using the Multiple Imputation method.

### Statistical Analysis of Demographic and Baseline Characteristics

In this study, descriptive statistics for demographic and pretreatment characteristics will be presented for each group. Continuous variables will be analyzed using either ANOVA or the Kruskal-Wallis test, depending on the data distribution. Categorical variables will be analyzed using the chi-square test or Fisher exact test; Fisher exact test will be applied when more than 25% of cells in a contingency table have an expected frequency of less than 5. If necessary, significant differences in baseline characteristics between groups will be adjusted for by incorporating these characteristics as covariates in the efficacy variable analysis.

### Study Protocol Modifications

Any modifications to the study protocol require prior approval from the institutional review board. This clinical trial will adhere to the approved protocol unless deviations are necessary to eliminate immediate risks to participants.

### Adverse Events

In this clinical trial, adverse events are defined as any harmful or unintended signs, symptoms, or diseases occurring in participants. It should be noted that not all such events necessarily have a causal relationship with the intervention. Serious adverse events include death or life-threatening situations, hospitalization or prolongation of existing hospitalization, permanent or significant disability or impairment, congenital anomalies or defects in a fetus, or other medically significant conditions such as drug dependency or abuse. However, not all adverse events are regarded as serious.

From the start of the intervention, any adverse events will be documented in detail, including the date of onset and resolution, the severity and outcome, the actions taken in relation to the intervention and the causal relationship with the intervention, any other suspected treatments or medications, and whether treatment for the adverse event was administered. During the study period, any serious adverse events must be reported by the research team to the principal investigator within 24 hours. The principal investigator is required to report all serious adverse events to the institutional review board in accordance with clinical trial regulations.

### Dropout

In this study, dropout refers to the termination of participation before completing all phases of the study. Researchers can stop treatment and observation and withdraw the participant, or participants can voluntarily withdraw at any time. A participant may be considered dropped out under the following circumstances:

1. If it is discovered after screening that the participant does not meet the inclusion or exclusion criteria.
2. If a systemic disease not identified during pretreatment examinations, is discovered.
3. If the participant experiences a serious adverse event and the researcher determines that continued participation is inappropriate.
4. If an adverse event is severe enough that the researcher deems continued participation to be inappropriate.
5. If symptoms worsen and the researcher determines that other treatment is necessary.
6. If the participant withdraws consent or requests to stop participation.
7. If the participant does not visit or cannot be followed up.
8. If the researcher determines that continuation of the clinical trial is inappropriate for any other reason.

### Discontinuation

In the following cases, this study can be discontinued early after consultation with the sponsoring organization.

1. If an unexpected and unacceptable risk is identified in a participant.
2. If moderate or severe adverse events considered related to the intervention occur in more than 25% of the participants.
3. If not all participants are enrolled by the end of the recruitment period.

### Termination

This clinical trial will officially terminate once the target number of participants has been enrolled and data completeness and integrity have been confirmed.

### Ethical Considerations

The study protocol, consent forms, participants’ information sheets, and all other documentation provided to the participants have received approval from the Institutional Review Board of Daejeon University Daejeon Medical Center (DJDSKH-22-BM-19). This clinical trial has been registered with the Clinical Information Services as of April 21, 2023 (identifier: KCT0008378). All participants will be thoroughly informed about the details of the clinical trial. The consent of participants will be obtained in accordance with the ethical principles and standards based on the Declaration of Helsinki. All participants will have the right to withdraw their consent voluntarily at any time, and all personal information—such as name, gender, and age—will be anonymized or deidentified to ensure that individuals cannot be identified. Participants will receive a nominal transportation allowance (less than US $300) as compensation.

## Results

**Figure 4:**
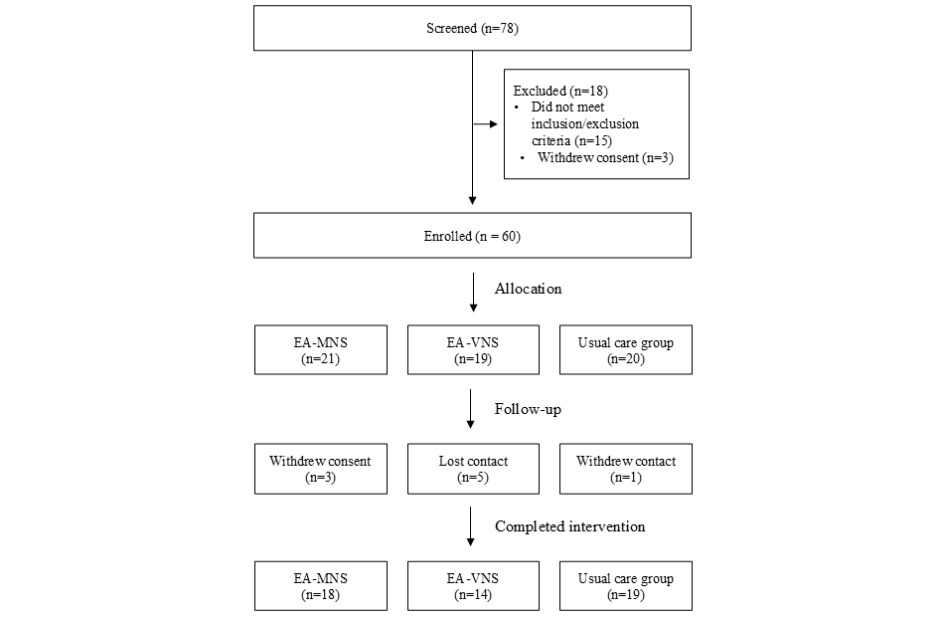
CONSORT (Consolidated Standards of Reporting Trials) flowchart. EA-MNS: electroacupuncture-median nerve stimulation; EA-VNS: electroacupuncture-vagus nerve stimulation;.

This study was funded in December 2022. The first participant was enrolled on December 15, 2022, and the final participant completed the intervention on November 22, 2024. A total of 60 participants were enrolled, and 51 participants completed the intervention. Data collection was conducted from December 13, 2022, to November 22, 2024. Data analysis is currently underway, and the results are expected to be published in 2025. The CONSORT (Consolidated Standards of Reporting Trials) flowchart depicting the study process is presented in Figure 4.

## Discussion

### Overview

Acupuncture has been shown to improve various mental health conditions, including depression and insomnia [34,35], and has also demonstrated effectiveness in the treatment of anxiety disorders [13]. However, electroacupuncture for anxiety disorders has primarily been investigated in animal studies, with a limited number of clinical trials reported to date [13]. Therefore, further clinical research is necessary to establish the clinical efficacy of electroacupuncture for anxiety disorders.

Some studies have reported that patients with anxiety disorders exhibit increased activity in the motor areas and motor networks of the brain [36,37]. This finding suggests that modulation of cerebral motor area functions could potentially regulate anxiety symptoms. In this study, the frequency of the median nerve is set at 12 Hz, based on previous studies suggesting that 12 Hz median nerve stimulation modulates the function of the brain’s motor areas and improves symptoms. In this study, the frequency of median nerve stimulation is set at 12 Hz, based on a previous study. Studies [38,39] demonstrated that 12 Hz median nerve stimulation is effective in modulating the function of the brain’s motor areas and improving symptoms. Similarly, the frequency of vagus nerve stimulation is set at 100 Hz to maximize the brain functions based on a previous study that found 100 Hz stimulation to be effective in increasing activity in the primary sensory cortex, premotor cortex, insular cortex, brainstem, and cerebellum [40].

### Strengths

To ensure the reliability of the study results, we used the SCID-5 to minimize the inclusion of participants who do not meet the diagnostic criteria for anxiety disorders. SCID-5 is a widely validated diagnostic tool with high reliability and validity in identifying anxiety disorders [41].

Anxiety symptom severity has been strongly linked to a decline in quality of life [42]. To assess the broader impact of treatment, this study includes the WHOQOL-BREF, enabling an evaluation of whether electroacupuncture improves not only anxiety symptoms but also patients’ overall quality of life.

Furthermore, we will use HRV to elucidate the physiological mechanisms of electroacupuncture in treating anxiety, particularly focusing on the regulation of the autonomic nervous system. HRV analysis can offer a noninvasive and objective assessment of the autonomic nervous system’s response to electroacupuncture, potentially evaluating the underlying mechanisms.

### Limitations

Although previous studies have suggested that neural stimulation may influence brain function in individuals with anxiety disorders [36,37], this study does not incorporate neurophysiological assessments, such as functional magnetic resonance imaging or electroencephalography, to directly evaluate these effects. As a result, this study is unable to determine whether electroacupuncture modulates brain activity at a neurophysiological level.

In addition, this study consists of an 8-week treatment period without posttreatment follow-up, focusing exclusively on the short-term effects of electroacupuncture. Future studies should investigate the long-term efficacy of electroacupuncture, its sustained effects after treatment cessation, and the potential for symptom relapse.

### Conclusion

The purpose of this clinical trial is to evaluate the efficacy and safety of electroacupuncture-neurostimulation in the treatment of anxiety disorders compared to a control group. We expect that electroacupuncture, through the stimulation of the median and vagus nerves, could become a novel therapeutic option for anxiety disorders.

## Appendix

Multimedia Appendix 1 STRICTA (Standards for Reporting Interventions in Clinical Trials of Acupuncture) checklist.

## Abbreviations

ANCOVA: analysis of covariance
BAI: Beck Anxiety Index
BDI-II: Beck Depression Inventory-II
CONSORT: Consolidated Standards of Reporting Trials
C-SSRS: Columbia Suicide Severity Rating Scale
DSM-5: Diagnostic and Statistical Manual of Mental Disorders (Fifth Edition)
EA-MNS: electroacupuncture-median nerve stimulation
EA-VNS: electroacupuncture-vagus nerve stimulation
EQ: empathy quotient
HAM-A: Hamilton Anxiety Rating Scale
HRV: heart rate variability
LSAS-SR: Leibowitz Social Anxiety Scale-Self Report
MMRM: Mixed-effect Model Repeated Measure
PDSS: The Panic Disorder Severity Scale
PHQ-15: Patient Health Questionnaire-15
PPS: Per Protocol Set
PSS: Perceived stress scale
PSWQ: Penn State Worry Questionnaire
SAS: Safety Analysis Set
SCID-5: Structured Clinical Interview for DSM-5
SPIRIT: Standard Protocol Items: Recommendations for Interventional Trials
STRICTA: Standards for Reporting Interventions in Clinical Trials of Acupuncture
WHOQOL-BREF: World Health Organization Quality of Life Assessment Instrument abbreviated version
