# Supplementary figures and images for: Efficacy and Safety of Electroacupuncture Through Nerve Stimulation in Patients With Anxiety Disorders: Protocol for a Randomized, Assessor-Blind, Three-Arm, Parallel-Group Clinical Trial
Source: JMIR Res Protoc. 2025 Jul 21;14:e68166. doi: 10.2196/68166 (PMC12322611; doi:10.2196/68166)

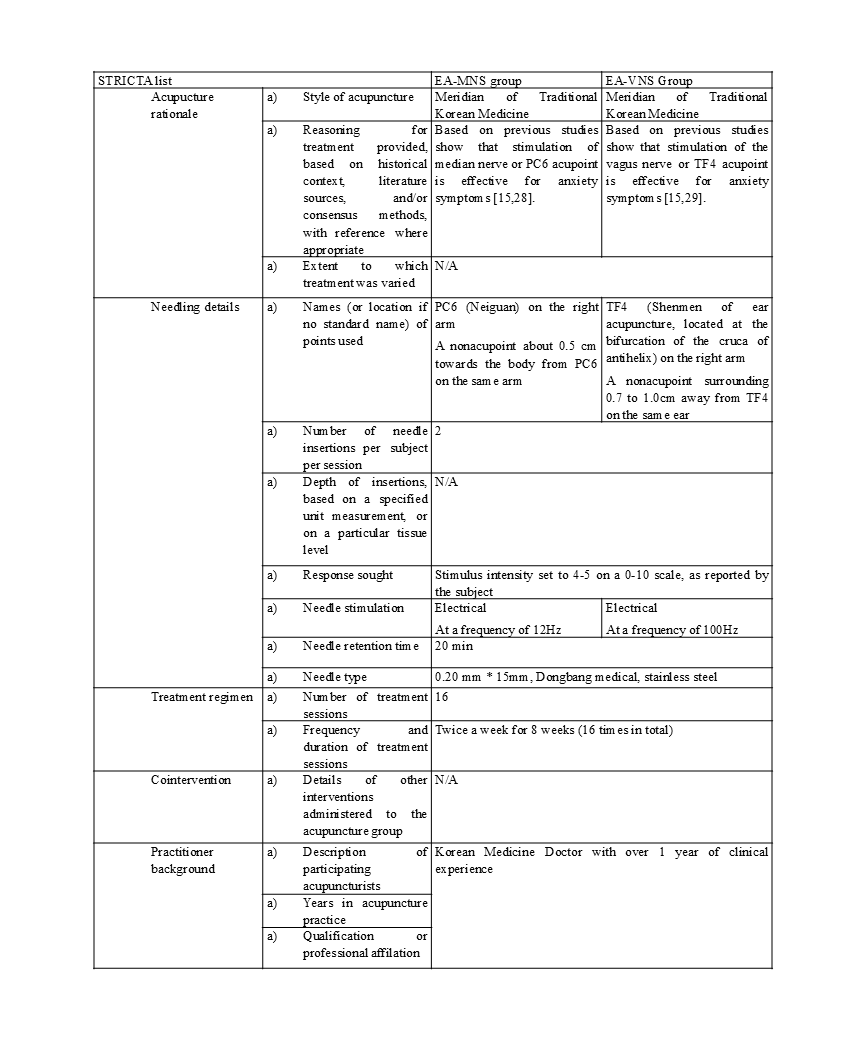

Supplement: Multimedia Appendix 1 [file resprot_v14i1e68166_app1.png]
